# Knockdown of lysine (K)-specific demethylase 2B KDM2B inhibits glycolysis and induces autophagy in lung squamous cell carcinoma cells by regulating the phosphatidylinositol 3-kinase/AKT/mammalian target of rapamycin pathway

**DOI:** 10.1080/21655979.2021.2005931

**Published:** 2021-12-07

**Authors:** Zhonghai Xie, Hongwei Li, Jin Zang

**Affiliations:** Department of Thoracic Surgery, Huzhou Central Hospital, Huzhou, Zhejiang Province, China

**Keywords:** KDM2B, glycolysis, autophagy, lung squamous cell carcinoma, PI3K/Akt/mTOR pathway

## Abstract

Lung squamous cell carcinoma (LUSC) is a subtype of non-small cell lung cancer with poor prognosis. This study aimed to explore the role of KDM2B in the development of LUSC. The results of this study demonstrated that KDM2B was upregulated in LUSC tissues and cell lines. Knockdown of KDM2B reduced cell viability and colony forming ability in SK-MES-1 and NCI-H520 cells. KDM2B inhibition reduced glucose consumption, lactate production, ATP level, and also downregulated the expression of LDHA and GLUT1. KDM2B knockdown decreased the protein expression of LC3-I and p62, and increased LC3-II and Beclin-1. Furthermore, KDM2B silencing inhibited the phosphorylation of AKT, mTOR and P70S6K. KDM2B knockdown led to reduced tumor size in mouse model. In conclusion, KDM2B is upregulated in LUSC tissues and cell lines. KDM2B silencing inhibits glycolysis and promotes autophagy through inactivation of the PI3K/Akt/mTOR signaling pathway.

## Introduction

Lung squamous cell carcinoma (LUSC) belongs to a subtype of non-small cell lung cancer (NSCLC) with distinctive histological features. LUSC accounts for 25–30% of NSCLC cases [1]. LUSC is highly associated with tobacco smoking, older age and is usually diagnosed as advanced disease [1,2]. In the past decades, targeted therapy and immunotherapy have been widely used in the treatment of NSCLC. However, these novel treatments were mainly effective in lung adenocarcinoma but not LUSC [3]. There has not been much progress in LUSC treatment. Although anti-VEGF therapy has been investigated in LUSC, to date there is no personalized therapy for LUSC [3]. Thus, the mechanisms leading to LUSC should be investigated to identify potential target drugs for personalized therapy of LUSC.

Comprehensive genomic sequencing has identified several potential gene mutations and signaling pathways that may be involved in LUSC, such as PI3KCA mutation, glycolysis and autophagy [3–5]. It is known that glycolysis-related genes are significantly enriched in LUSC and autophagy-related genes have been associated with the prognosis of LUSC [4,5]. Therefore, glycolysis and autophagy are useful indicators to predict the survival of LUSC patients. The phosphatidylinositol 3-kinase (PI3K)/AKT/mammalian target of rapamycin (mTOR) pathway is a well-known signaling pathway involved in cancer development and progression, including LUSC [6], indicating that this pathway may be a potential therapeutic target for LUSC patients.

Lysine (K)-specific demethylase 2B (KDM2B) is a family member of JmjC and is ubiquitously expressed in the nucleus [7]. KDM2B promotes cell proliferation, migration and prevents cell differentiation [7]. Overexpression of KDM2B was observed in breast cancer, lung cancer and gastric cancer [7], suggesting that KDM2B regulates cancer development. For example, KDM2B was found to promote cell proliferation in triple negative breast cancer through suppressing the transcription of p15INK4B, p16INK4A, and p57KIP2 [8]. In pancreatic ductal adenocarcinoma, KDM2B promotes cancer progression through regulating hippo pathway [9]. These results indicate that KDM2B might contribute to the progression of cancer. However, to date, no study has reported the role of KDM2B in LUSC. We hypothesized that KDM2B promotes the development and progression of LUSC. Thus, the purpose of this study was to explore the role and mechanism of KDM2B in the development of LUSC, aiming to identify a new therapeutic target for LUSC.

## Methods

### TCGA database and human sample collection

The expression data of KDM2B in LUSC patient tumor tissues (n = 503) and normal tissues (n = 52) were downloaded from The Cancer Genome Atlas (TCGA, https://www.cancer.gov/about-nci/organization/ccg/research/structural-genomics/tcga). Human LUSC tissues and their adjacent normal tissues (n = 30) were collected during surgery at Huzhou Central Hospital. All patients have provided the written informed consents and agreed for sample collection and data publication. This research project has been approved by the Medical Ethics Committee of Huzhou Central Hospitala and complied with the Declaration of Helsinki [10].

### Cell culture and cell treatment

Human normal lung epithelial cell line (BEAS-2B cells) and lung squamous cell carcinoma cell lines (SK-MES-1 cells and NCI-H520 cells) were obtained from Wuhan Procell Life Science&Technology Co., Ltd (Procell, China). Minimum Essential Medium Eagle (MEM, Sigma-Aldrich, USA) culture medium was supplemented with 10% fetal bovine serum (FBS, Beyotime, China) and 1% Antibiotic Antimycotic Solution (100×, Sigma-Aldrich). All cells were incubated in culture medium in a humid incubator maintained at 37°C with 5% CO_2_.

KDM2B small interfering RNA (si-KDM2B) and its scrambled RNA (si-NC) were obtained from Thermo Fisher Scientific Inc. (Thermo Fisher, USA) and reconstituted in sterile H_2_O at the concentration of 1 mg/ml. Cell transfection was performed using Lipofectamine RNAiMAX Transfection Reagent (Thermo Fisher) according to the manufacturer’s instructions.

### MTT assay and cell colony formation assay

For MTT assay [11], 5 × 10^4^ cells/well of LUSC cells were cultured into sterile 24-well plates. After transfection with si-KDM2B or si-NC for 24 h, cells were transferred to 96-well plates. 20 μl of MTT solution (Beyotime, China) was added into each well and incubated for 4 h at 37°C. After that, 100 μl of Formazan solution was added to each well and the plates were further incubated for 4 h at 37°C. The optical density (OD) value at 490 nm was determined using a Multiskan SkyHigh Microplate Spectrophotometer (Thermo Fisher).

For cell colony formation assay [12], si-KDM2B or si-NC were transfected into LUSC cells. The transfected cells were then seeded into 6-well plates with 5 ml of culture medium containing 0.4% of agarose. The plates were incubated at 37°C for one week. After that, cells were fixed with Image-iT™ Fixative Solution (Thermo Fisher) and stained with 0.1% crystal violet (Beyotime). Cell colonies were photographed and the number of colonies formed was counted under a microscope (Olympus, Japan).

### Quantitative real-time polymerase chain reaction (qRT-PCR)

Relative mRNA expression was measured using qRT-PCR [13]. Briefly, total RNA was isolated from samples using a High Pure RNA Isolation Kit (Roche, Switzerland). A total of 200 ng RNA was reverse transcribed to cDNA. Relative expression of mRNA was determined using a OneTaq® RT-PCR Kit (BioLabs, USA) and calculated using 2^−ΔΔCT^ method. Primer sequences (Sigma-Aldrich) used in this study are as follows: KDM2B forward, 5’-TCTACGAGATCGAGGACAGGA-3’ and reverse, 5’-ACCAGCACATCTCATAGTAGAAGG-3’; and β-actin forward, 5’- CTCGACACCAGGGCGTTATG-3’ and reverse, 5’- CCACTCCATGCTCGATAGGAT-3’.

### Western blotting

Protein expression was determined using Western blotting [14]. Total proteins were extracted using RIPA Lysis and Extraction Buffer (Thermo Fisher). Pierce™ BCA Protein Assay Kit (Thermo Fisher) was used to measure the concentrations of protein. 5 μg of protein was loaded and separated by SDS-PAGE gel (BioRad, USA). The separated protein was then transferred onto the PVDF membranes (Merck KGaA, German). After that, membranes were blocked by 5% of fat-free milk for 1 h at room temperature and followed incubation with primary antibodies overnight at 4°C. Membranes were then probed with appropriate secondary antibodies for 2 h at room temperature. Finally, the protein signals were detected using the Pierce™ ECL Western blotting Substrate (Thermo Fisher, USA). Primary antibodies (Abcam) used in this study are as follows: KDM2B (ab5199, 1:1500 dilution), LDHA (ab52488, 1:2000 dilution), GLUT1 (ab115730, 1:1000 dilution), LC3-I (ab244175, 1:1000 dilution). LC3-II (ab192890, 1:1500 dilution), p62 (ab207305, 1:1000 dilution), Beclin-1 (ab210498, 1:2000 dilution), p-Akt (ab38449, 1:500 dilution), Akt (ab8805, 1:800 dilution), p-mTOR (ab109268, 1:1000 dilution), mTOR (ab32028, 1:1500 dilution), p-P70S6K (MA5-15202, 1:1000 dilution, Thermo Fisher), P70S67 (A300-510A, 1:500 dilution, Thermo Fisher), GAPDH (ab8245, 1:3000 dilution) and β-actin (ab8227, 1:5000 dilution).

### Measurement of glucose consumption, lactate production and adenosine-triphosphate (ATP) level

Glucose consumption and lactate production were assessed using the Glucose Uptake Colorimetric Assay Kit (Merck KGaA) and L-Lactate Assay Kit (Abcam, UK), respectively. ATP level was examined using the ATP Assay Kit (Abcam).

### In vivo *xenograft growth*

Male C57BL/6 J mice (15 weeks old) were obtained from Jiangsu ALF Biotechnology Co., LTD. All mice were kept under 12 h light/12 h dark cycle at 25 ± 2°C and the humidity of the environment was 55 ± 15% for 1 week. Mice were fed with regular mouse food and drinking water. The *in vivo* xenograft model was constructed in compliance with the ethical standards under a protocol approved by the Experimental Animal Ethics Committee of Hangzhou Yingyang Biomedical R&D Center and were executed in accordance with the Care and Use of Laboratory Animals published by the US National Institutes of Health (No. 85–23, 1996).

SK-MES-1 cells were transfected with si-KDM2B or si-NC for 24 h. After that, 5 × 10^6^ transfected SK-MES-1 cells were resuspended in normal saline and then injected into the back of the mice (n = 6 mice/group). Mice were euthanized with an overdose of pentobarbital (100 mg/kg) after 14 days. The tumor size was measured.

### *Immunohistochemistry* [15]

Tumor tissues were collected and fixed with 4% paraformaldehyde overnight. The fixed tumor tissues were embedded in paraffin wax, cut into 5 µm sections and fixed on the glass slides. Deparaffinization and rehydration were performed using xylene and graded ethanol solutions, respectively. Antigen retrieval was conducted by boiling the tissue sections in 10 mM sodium citrate buffer (pH 6.0) for 5 minutes. The sections were then blocked with goat serum for 1 h and probed using primary antibody overnight at 4°C and then incubated with biotinylated goat anti-mouse antibodies for 2 h at room temperature. Finally, the sections were incubated with streptavidin conjugated to horseradish peroxidase and diaminobenzidine was used as the chromogen.

## Statistical analysis

All statistical tests were performed using GraphPad Prism 8.1.0 (GraphPad, USA). All data are presented as mean ± SD. Student’s t-test and one-way analysis of variance (ANOVA) were used to compare the differences between two groups, or multiple groups, respectively. *p* < 0.05 was considered statistically significant.

## Results

As aforementioned, the purpose of this study was to explore the role and mechanism of KDM2B in the development of LUSC, aiming to identify a new therapeutic target for LUSC. Thus, we examined the expression of KDM2B and its effects on glycolysis and autophagy in LUSC. The underlying mechanism was also investigated.

### KDM2B is upregulated in LUSC tumor tissues and cell lines

**Figure 1.**
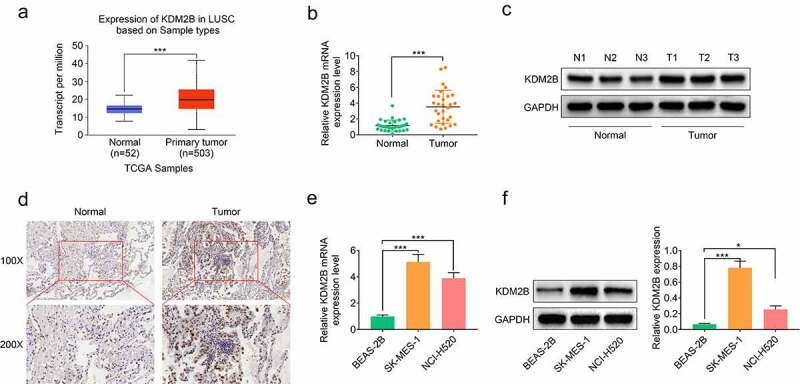
KDM2B is upregulated in LUSC tumor tissues and cell lines. (a) KDM2B expression is increased in primary tumor tissues based on analysis of the expression data derived from the TCGA database; (b) KDM2B transcript level is increased in LUSC patient tissues; (c) Western blot images show that KDM2B protein level is increased in LUSC patient tissues; (d) Immunohistochemistry images show higher KDM2B expression in LUSC patient tissues compared to normal tissues (IHC); (e) Histogram showing increased KDM2B transcript level in LUSC cells; (f) Increased protein expression of KDM2B in LUSC cells. *p < 0.05 versus (vs) normal or BEAS-2B; ***p < 0.005 vs normal or BEAS-2B. LUSC: Lung squamous cell carcinoma

Analysis of expression data obtained from TCGA database showed that KDM2B was upregulated in primary tumor tissues (Figure 1a). KDM2B expression was increased at both mRNA and protein levels in LUSC patient tumor tissues compared with their adjacent normal tissues (Figure 1b, c and d). KDM2B expression was also found to be higher in LUSC cell lines (SK-MES-1 and NCI-H520) compared to human normal lung normal BEAS-2B cell line, at both mRNA and protein levels (Figure 1e and f).

### KDM2B knockdown inhibits cell proliferation and glycolysis, and induces autophagy in lung squamous cell cancer cells

**Figure 2.**
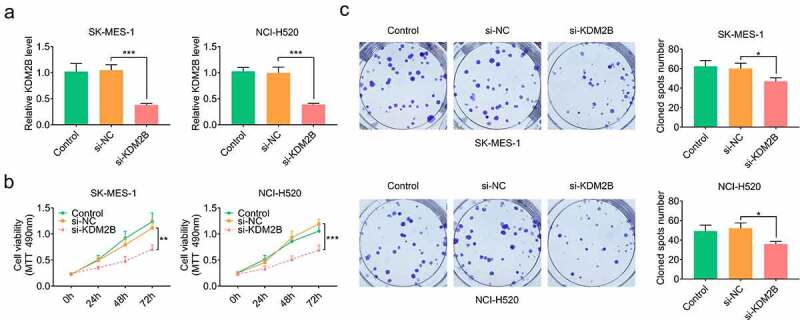
KDM2B knockdown inhibits cell proliferation in LUSC cell lines. (a) KDM2B expression is decreased in SK-MES-1 and NCI-H520 cells; (b) Cell viability is inhibited in SK-MES-1 and NCI-H520 cells transfected with si-KDM2B; (c) The number of cell colonies formed is reduced by si-KDM2B in SK-MES-1 and NCI-H520 cells. *p < 0.05 vs si-NC; **p < 0.01 vs si-NC ***p < 0.005 vs si-NC. si-KDM2B: small interfering RNA (siRNA) targeting KDM2B; si-NC: scrambled non-targeting control siRNA

KDM2B silencing by si-KDM2B transfection significantly downregulated the expression of KDM2B in LUSC cells (Figure 2a). Results from the MTT assays demonstrated that cell viability was significantly reduced by si-KDM2B compared with si-NC and control group in LUSC cells (Figure 2b). A significant reduction in the colony forming ability was also observed in LUSC cells transfected with si-KDM2B (Figure 2c).

**Figure 3.**
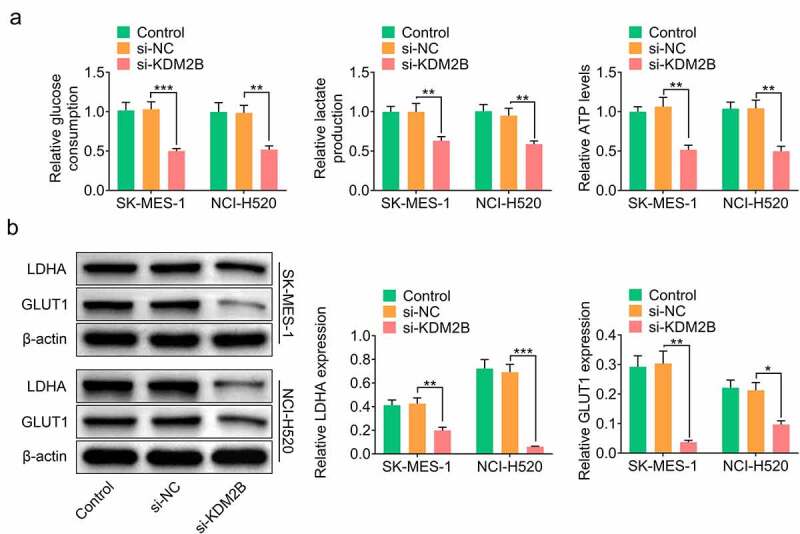
KDM2B silencing represses glycolysis in LUSC cell lines. (a) KDM2B knockdown reduces glucose consumption, lactate production and ATP level in SK-MES-1 and NCI-H520 cells; (b) KDM2B silencing inhibits the protein expression of LDHA and GLUT1 in SK-MES-1 and NCI-H520 cells. *p < 0.05 vs si-NC; **p < 0.01 vs si-NC ***p < 0.005 vs si-NC

**Figure 4.**
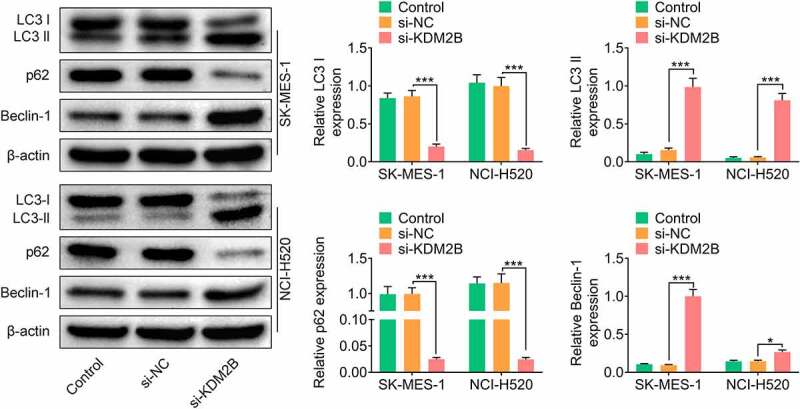
Knockdown of KDM2B inhibits autophagy in LUSC cell lines. KDM2B silencing decreases the protein expression of LC3-I and p62 and increases the protein expression of LC3-II and Beclin-1 in SK-MES-1 and NCI-H520 cells. *p < 0.05 vs si-NC; **p < 0.01 vs si-NC ***p < 0.005 vs si-NC

In addition, KDM2B silencing by si-KDM2B reduced glucose consumption, lactate production and ATP level in LUSC cells (Figure 3a). The protein levels of lactic dehydrogenase A (LDHA) and glucose transporter type 1 (GLUT1) were significantly reduced in LUSC cells transfected with si-KDM2B compared to control and si-NC groups (Figure 3b). The protein expression of microtubule-associated protein light chain 3-I (LC3-I) and p62 was downregulated, whereas LC3-II and Beclin-1 were upregulated in LUSC cells transfected with si-KDM2B compared to control and si-NC groups (Figure 4).

### KDM2B regulates the PI3K/Akt/mTOR signaling pathway

**Figure 5.**
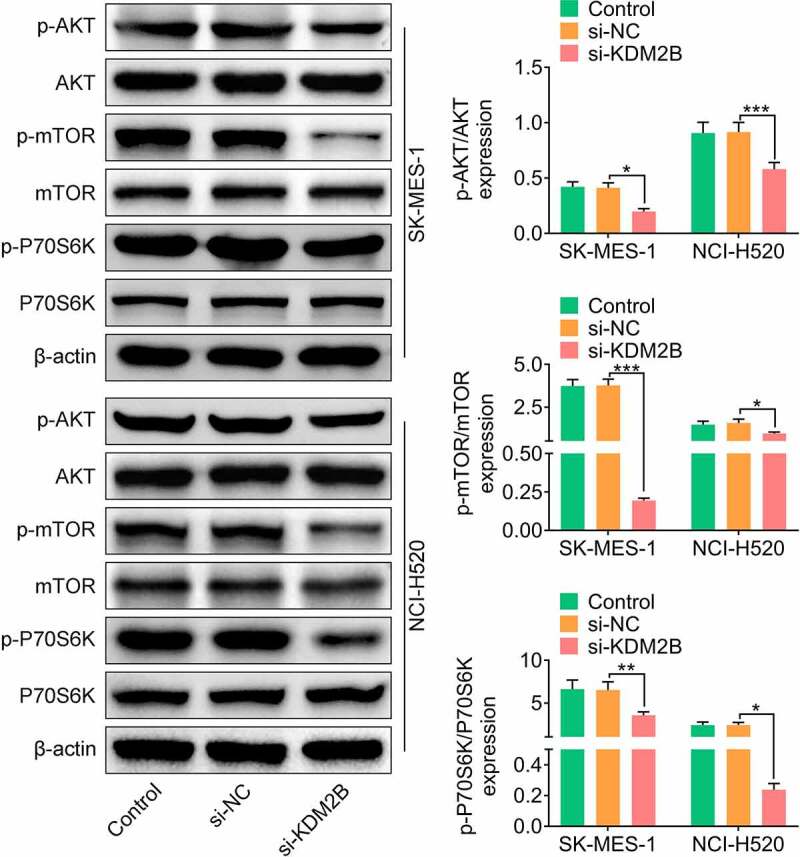
KDM2B regulates the PI3K/Akt/mTOR signaling pathway. KDM2B knockdown inhibits the phosphorylation of AKT, mTOR and P70S6K in SK-MES-1 and NCI-H520 cells. *p < 0.05 vs si-NC; **p < 0.01 vs si-NC ***p < 0.005 vs si-NC

Next, the effect of KDM2B silencing on the PI3K/Akt/mTOR signaling pathway was investigated. In LUSC cells transfected with si-KDM2B, a significant reduction in the phosphorylation levels of AKT, mTOR and P70S6K was observed compared to control and si-NC groups (Figure 5).

### Knockdown of KDM2B represses tumor growth in xenograft tumor model

**Figure 6.**
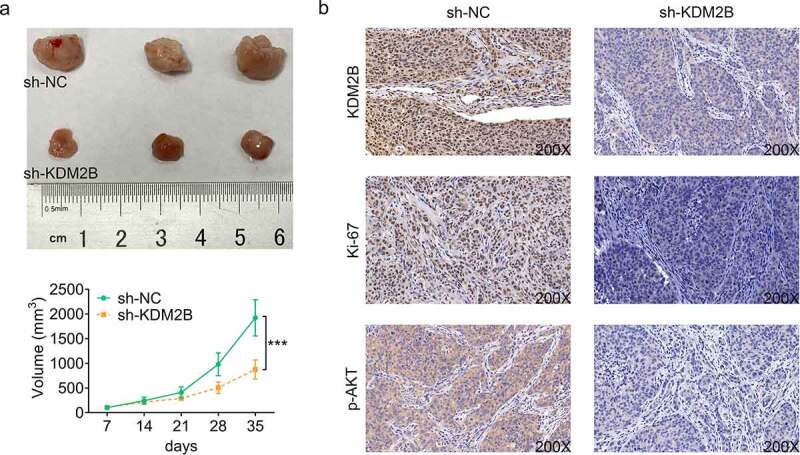
Knockdown of KDM2B represses tumor growth in mouse model. (a) Knockdown of KDM2B reduces the tumor size; (b) KDM2B silencing inhibits the protein expression of KDM2B, Ki67 and phosphorylated AKT. ***p < 0.005 vs si-NC

Next, we sought to investigate the effect of KDM2B silencing on tumor size in xenograft tumor model. Our results showed that KDM2B knockdown led to a significant reduction in the tumor volume in mice transfected with si-KDM2B (Figure 6a). As shown in Figure 6b, the expression of KDM2B was reduced by si-KDM2B in tumor tissues, indicating the efficiency of si-KDM2B in depleting KDM2B (Figure 6b). A decrease in Ki67 and phosphorylated AKT was also observed in tumor tissues transfected with si-KDM2B (Figure 6b).

## Discussion

In the past years, the clinical outcomes of lung adenocarcinoma have been greatly improved with the introduction of targeted therapy and immunotherapy. However, the prognosis and survival of LUSC patients remain elusive [3]. Thus, there is an urgent need to understand the mechanisms leading to the pathogenesis of LUSC in order to identify potential therapy or treatment for LUSC. In this study, KDM2B was found to be overexpressed in LUSC tissues and cell lines. Downregulation of KDM2B using RNA silencing method inhibited LUSC cell proliferation and also reduced glycolysis and induced autophagy in LUSC cell lines through inhibiting the activation of PI3K/Akt/mTOR signaling pathway. Further experiment found that knockdown of KDM2B inhibited tumor growth in mouse model. Thus, KDM2B promoted the progression of LUSC, providing a potential new therapeutic target for the treatment of LUSC.

Glycolysis is a metabolic pathway that breaks down glucose or glycogen to produce ATP [16]. It is reported that glycolysis-related genes are upregulated in over 70% of cancers to increase the ATP production, which facilitates cancer cell proliferation [16]. Several tumor-related genes have been identified to regulate tumor growth through modulating glycolysis [17], but the mechanism remains unclear. KDM2B has been reported to increase glucose metabolism in type 1 diabetes [18]. In this study, si-KDM2B reduced glucose consumption, lactate production and inhibited the production of ATP in LUSC cell lines, indicating that KDM2B induces glycolysis to provide more energy to cell growth in LUSC. The protein expression of GLUT1 and LDHA was reduced following KDM2B knockdown, further confirming that KDM2B is involved in glycolysis.

Autophagy is a process that degrades proteins and organelles in response to cellular stress [16,19]. Basal autophagy is considered as a tumor-suppressor in the early stage of cancer [20]. Glycolysis regulates autophagy in cancer cells and suppression of glycolysis enhances autophagy [16]. Therefore, this study also examined autophagy after knockdown of KDM2B. The results demonstrated that inhibition of KDM2B enhanced autophagy in LUSC cell lines, which is consistent with previous findings [21]. Taken together, these data suggest that KDM2B promotes glycolysis and inhibits autophagy in LUSC.

The PI3K/Akt/mTOR pathway is a well-known signaling pathway involved in cancer development and progression [6]. In the present study, KDM2B silencing inhibited the phosphorylation of AKT, mTOR and P70S6K, indicating that PI3K/Akt/mTOR signaling pathway mediates KDM2B-induced glycolysis and suppression of autophagy. Inhibition of the PI3K/Akt/mTOR signaling pathway has been investigated as a therapeutic target in cancer treatments [6]. Several inhibitors have been approved by the US Food and Drug Administration (FDA), including alpelisib (PI3K inhibitor) [22] and everolimus (mTOR inhibitor) in breast cancer [23]. Several clinical trials are ongoing to investigate the efficacy of PI3K/Akt/mTOR pathway inhibitors in LUSC [6]. Hopefully more targeted drugs will become available to improve the clinical outcomes of LUSC patients in the future.

## Conclusion

In conclusion, the findings from this study demonstrate that KDM2B is overexpressed in LUSC tissues and cell lines. Knockdown of KDM2B inhibits cell proliferation i*n vitro* and tumor growth *in vivo* in LUSC. Furthermore, inhibition of KDM2B represses glycolysis and induces autophagy through inactivation of the PI3K/Akt/mTOR signaling pathway, providing a new therapeutic target for the treatment of LUSC in the future.

## Data Availability

All data generated or analyzed during this study are included in this published article.

## Highlight

- KDM2B was upregulated in LUSC tumor tissues and cell lines.
- Knockdown of KDM2B inhibited cell proliferation and glycolysis, and induced autophagy in lung squamous cell cancer cells.
- KDM2B regulated the PI3K/Akt/mTOR signaling pathways.
- Knockdown of KDM2B repressed the tumor growth in xenograft tumor model.
